# Proactive interference in aging: A model-based study

**DOI:** 10.3758/s13423-019-01671-0

**Published:** 2019-12-03

**Authors:** Kim Archambeau, Birte Forstmann, Leendert Van Maanen, Wim Gevers

**Affiliations:** 1 Center for Research in Cognition and Neurosciences, ULB Neuroscience Institute, Université Libre de Bruxelles, Brussels, Belgium; 2 Department of Psychology, University of Amsterdam, Amsterdam, The Netherlands

**Keywords:** Proactive interference, Aging, Diffusion decision model, Inhibition, Short-term memory

## Abstract

Proactive interference occurs when previously learned information interrupts the storage or retrieval of new information. Congruent with previous reports, traditional analyses dealing with response times and error rates separately have indicated an increase in sensitivity to proactive interference in older adults. We reanalyzed the same data using diffusion decision model (DDM). Such models enable a more fine-grained interpretation concerning the latent processing mechanisms underlying performance. Now a different picture emerged. The DDM results showed that older adults needed more evidence than young adults before responding. The results also clearly indicated that peripheral processes (encoding time and motor execution), as well as recognition memory, decline with age. However, the drift rates, reflecting proactive interference, were similar, suggesting—contrary to earlier reports—that the inhibitory processes observed with this paradigm remain intact in older adults.

Proactive interference (PI) occurs when information previously stored disturbs the learning or remembering of new information. PI negatively affects performance in short-term memory, which is crucial for normal cognitive processing (e.g., Conway & Engle, 1994; Engle, Conway, Tuholski, & Shisler, 1995). Given that PI is considered one of the principal sources of forgetting in short-term memory (Jonides et al., 2008), a better understanding of age-related changes in PI constitutes a major objective in cognitive psychology.

PI in short-term memory is frequently investigated using the recent-probes task (Monsell, 1978). This is a recognition task in which participants memorize a set of items (the target set; Fig. 1A). After a short retention interval, a probe is presented, and participants have to decide whether it was one of the target set. This probe can be either positive, because it matches a member of the target set, or negative because not matching any member of the target set. Critically, although some negative probes were not presented recently (*non-recent* negative probes), other negative probes had been presented in the preceding target set (*recent* negative probes), inducing PI. PI experienced for recent negative probes hurts performance by slowing response times (RTs) and impairing rejection of these probes (for a review, see Jonides & Nee, 2006).

**Fig. 1:**
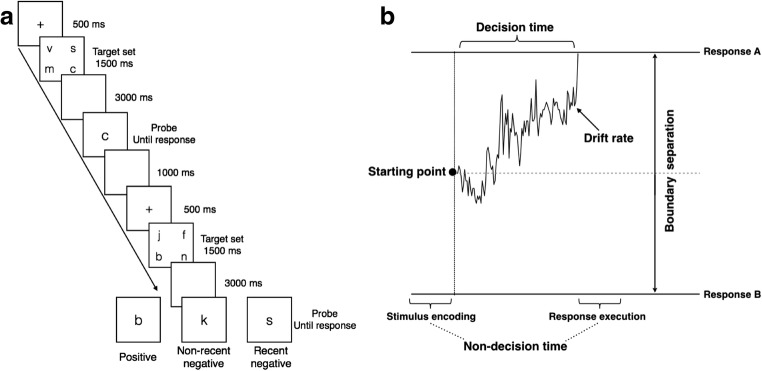
Schematic representations of the recent-probes task (A) and the diffusion decision model (B)

Regarding the effects of aging, it has repeatedly been reported that older adults have more difficulty resolving PI in short-term memory (e.g., Jonides et al., 2000; Manard, Carabin, Jaspar, & Collette, 2014). Specifically, the difference in performance between recent negative and non-recent negative probes was observed to be larger for older than for younger adults, suggesting an age-related increase in sensitivity to PI. Increased PI with age has previously been observed with either accuracy (e.g., Loosli, Rahm, Unterrainer, Weiller, & Kaller, 2014), RTs (e.g., Manard et al., 2014), or both (Pettigrew & Martin, 2014). Inhibition is assumed to play a role in the resolution or reduction of interference, by suppressing no-longer-relevant information that has already entered short-term memory (e.g., Friedman & Miyake, 2004; Lustig, Hasher, & Zacks, 2007). It is therefore possible that the increase of sensitivity to PI with age results from less efficient inhibitory processes in older than in younger adults (e.g., Jonides et al., 2000). This is consistent with the inhibitory deficit theory, arguing for a general decline across different types of inhibitory processes with age (Hasher & Zacks, 1988; Lustig et al., 2007). This theory proposes that older adults have difficulties preventing irrelevant information from entering or remaining in the focus of attention (Hasher & Zacks, 1988; Lustig et al., 2007). Indeed, deficient inhibitory processes in older adults have been observed across a wide range of cognitive tasks (for a review, see Hasher, Zacks, & May, 1999). However, not all cognitive tasks involving inhibition reveal such age-related deficiencies (e.g. Collette, Schmidt, Scherrer, Adam, & Salmon, 2009), suggesting that age-related inhibitory deficiencies may be specific to the inhibitory task at hand. Furthermore, even if older adults underperform in a given inhibitory task, this does not necessarily imply that the inhibitory process itself is deficient. Other functions, such as a decline in sensory processing or in motor execution time, can also contribute to lower performances (e.g., Burke & Osborne, 2007). For instance, Rabaglia and Schneider (2016) found worse suppression of irrelevant information in older than in younger participants. However, this difference disappeared when visual filters were applied, such that the contrast sensitivity of the targets was equal in both groups.

The relative contribution of these additional processes and how they are affected by age can be studied using the diffusion decision model (DDM). DDM analyses use both RTs and accuracy data to infer the most likely combination of parameters to have generated the data. These parameters are associated with psychological interpretations. Specifically, the DDM assumes that noisy information for each choice alternative is accumulated over time until a predefined boundary is reached, after which the response associated with this boundary is executed (Ratcliff, 1978; Ratcliff & McKoon, 2008). The standard DDM has four main parameters: *drift rate*, *boundary separation*, *starting point*, and *nondecision time* (Fig. 1B). *Drift rate* is the average rate of information accumulated per unit of time and therefore provides a measure of performance (processing efficiency). *Boundary separation* is defined by the amount of information needed to make a decision and reflects the level of caution. The *starting point* represents a predecision response bias for one or the other choice alternative. Finally, the *nondecision time* is a measure of peripheral processes such as stimulus encoding and response execution (e.g., Ratcliff, 1978; Ratcliff & McKoon, 2008). Because the parameter values might not be identical from trial to trial, DDM also includes intertrial variabilities of the drift rate, starting point, and nondecision time as additional parameters (for more details about the DDM and its application, see Ratcliff & McKoon, 2008).

Previous applications of DDM in the domain of aging (e.g., Ratcliff, Thapar, & McKoon, 2004, 2010) have typically shown an increase in nondecision time with age, meaning that older adults need more time to encode the stimuli and to execute motor responses than do young adults. Additionally, it was observed that older adults adopted a stronger level of caution, reflected by a higher boundary separation for older than for younger adults. Importantly, no decline with age was found in many cognitive processes (e.g., recognition memory), which was supported by similar drift rates between younger and older adults (e.g., Ratcliff et al., 2004).

The aim of the present study was to assess the psychological processes responsible for age-related differences in sensitivity to PI. To do so, a sample of younger and older adults performed the recent-probes task. We first compared the performance between age groups using traditional analyses on both mean RTs and error rates. Then, a DDM analysis was performed. Applying the DDM allowed us to determine whether the potential age differences observed with inferential analyses was due to a decline in cognitive processing or to other components of processing, such as peripheral processes or level of caution. Within the present task context, a possible main effect of age on drift rate is interpreted as reflecting difficulties in recognition memory (e.g., Ratcliff et al., 2010). Crucially, if the inhibitory processes underlying PI are affected by age, the difference in drift rate between recent negative and non-recent negative trials should increase with age.

## Method

### Participants

The participants were 25 young adults (*M* = 19.53 years, *SE* = 0.39; 19 women, 6 men) and 38 older adults (*M* = 71.95 years, *SE* = 1.50; 28 women, 10 men). We aimed for a final set of about 25 participants in each group. This number was based on earlier published studies on proactive interference, typically using this sample size (e.g., Loosli et al., 2014). More older adults were tested anticipating a higher rejection rate of participants in this age group following the exclusion criteria mentioned below. However, none of the older adults met these exclusion criteria, resulting in slightly imbalanced group sizes.

The young adults were undergraduates from the Université Libre de Bruxelles (ULB) and received course credits. The older adults were recruited through flyers and announcement on a website and received monetary compensation for their participation. The study protocol was approved by the local ethics committee (ULB, Faculty of Psychological and Educational Sciences). All participants were French-speakers and had normal or corrected-to-normal vision. The exclusion criteria were alcoholism, history of stroke, head trauma, epilepsy, metabolic or psychiatric disorders, or low level of education (at most primary education). The Montreal Cognitive Assessment (MoCA; Nasreddine et al., 2005) was used to discard older adults with potential risk of dementia (MoCA score > 26). Older adults with high depressive symptoms (score > 10) were excluded using the Geriatric Depression Scale (GDS; Yesavage et al., 1982). The older (*M* = 13.10 years, *SE* = 0.44) and young (*M* = 12.88 years, *SE* = 0.29) adults had similar years of education [*t*(61) = 0.38, *p* = .708]. Participants were tested at the university (32 participants) or at home (31 participants) in one session of approximately 60 min.

### Material and procedure

Participants performed the recent-probes task, during which they had to decide whether a probe was part of the target set that had previously been presented (Fig. 1A). A trial started with a fixation point (500 ms), followed by the target set (1,500 ms). The target set consisted of four lowercase consonants and was followed by a delay of 3,000 ms. Next, a single consonant probe was presented. Participants had to indicate whether or not the probe belonged to the current target set by performing a left or a right key press. After a response, the next trial was initiated, with an intertrial interval of 1,000 ms. On half of the trials, the probe required a positive response, because it was a member of the target set (positive trials). On the other half, the probe required a negative response, because the probe was not a member of the target set (negative trials). Two types of negative trials were used: recent negative trials and non-recent negative trials. For the recent negative trials, the probe had been a member of the previous target set. For the non-recent negative trials, the probe had not been a member of either of the two previous target sets. The task included three blocks of 160 trials (total 480 trials), with 240 positive trials, 120 non-recent negative trials, and 120 recent negative trials. Half of the participants had to press the left button (A key) for positive trials and the right button (L key) for negative trials, whereas this response mapping was reversed for the remaining participants. The task was run on a 17-in. laptop computer using Matlab.

## Results

The RTs cutoffs were 200 and 7,000 ms, which excluded 0.28% of the data. A repeated measures analysis of variance (ANOVA) with condition (three levels: positive, non-recent negative, recent negative) as a within-subjects factor and age (two levels: young adults, older adults) as a between-subjects factor was performed separately on error rates and RTs (only correct trials). To assess the PI effect, the critical comparison was the difference in performance between the non-recent negative and recent negative conditions.

**Fig. 2:**
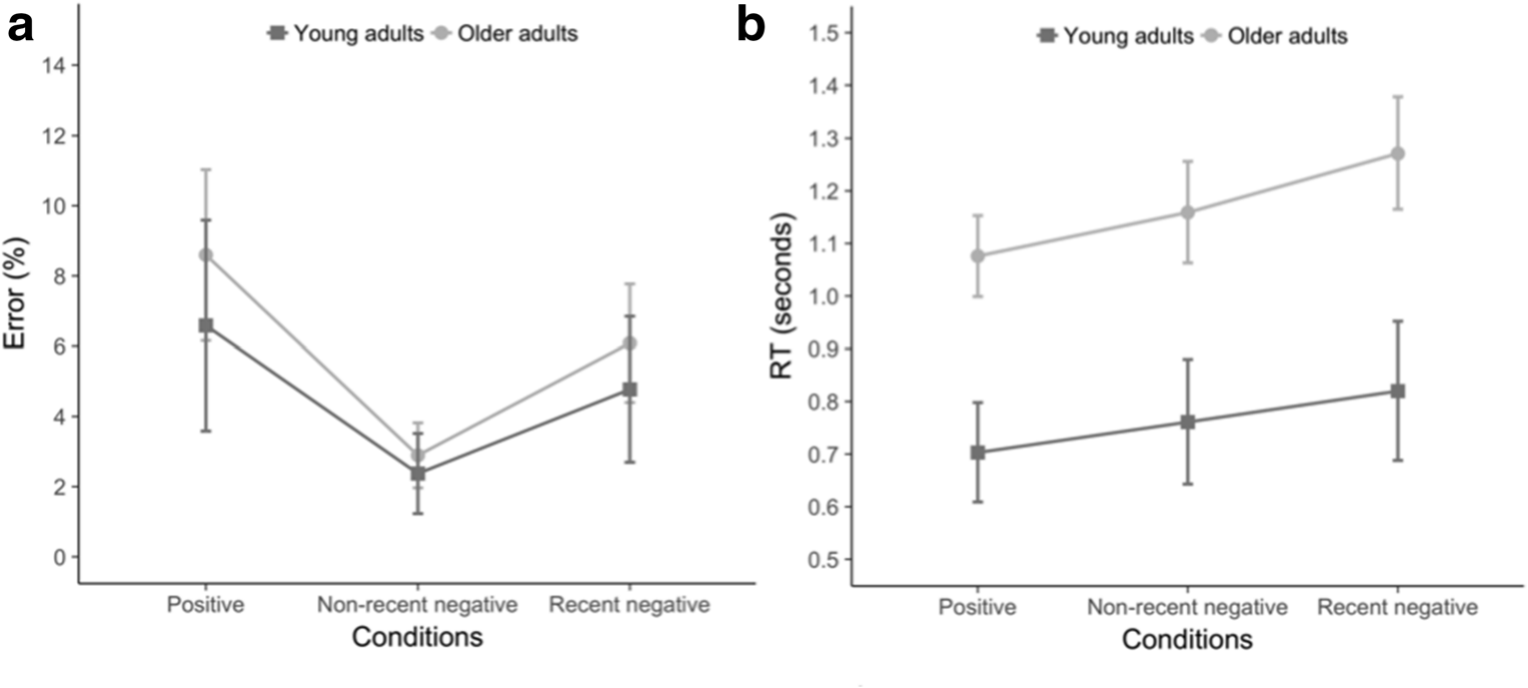
Behavioral results of the recent-probes task. Panels A and B depict the behavioral results for accuracy (A) and reaction times (B), averaged over participants and conditions for each age group (error bars represent 95% confidence intervals)

An analysis of error rates revealed a main effect of condition [*F*(2, 122) = 17.85, *p* < .001, *η*_p_^2^ = .23]. Planned comparisons showed that more errors were made in the recent negative than in the non-recent negative condition (*p* < .001), indicating a PI effect (recent negative condition, error rate = 5.43%, *SE* = 0.67; non-recent negative condition, error rate = 2.63%, *SE* = 0.37). The main effect of age was not significant [*F*(1, 61) = 1.49, *p* = .227, *η*_p_^2^ = .02]. The interaction between condition and age was not significant, either [*F*(2, 122) = 0.40, *p* = .669, *η*_p_^2^ = .007; Fig. 2A]. Planned comparisons indicated that the interaction between the PI effect and age did not reach significance [*F*(1, 61) = 0.75, *p* = .391, *η*_p_^2^ = .01].

The same analysis on RTs showed that the main effect of condition was significant [*F*(2, 122) = 42.38, *p* < .001, *η*_p_^2^ = .41]. Planned comparisons revealed that recent negative trials were responded to more slowly than non-recent negative trials (*p* < .001), indicating a significant PI effect (recent negative condition, mean RTs = 1,046 ms, *SE* = 42.35; non-recent negative condition, mean RTs = 960 ms, *SE* = 38.20). We found a significant effect of age [*F*(1, 61) = 32.01, *p* < .001, *η*_p_^2^ = .34], indicating that older adults (mean RTs = 1,169 ms, *SE* = 45.37) were globally slower than younger adults (mean RTs = 761 ms, *SE* = 55.94). The interaction between condition and age just failed to reach significance [*F*(2, 122) = 2.77, *p* = .066, *η*_p_^2^ = .04; Fig. 2B]. Importantly, planned comparisons showed that the PI effect significantly interacted with the factor age [*F*(1, 61) = 7.95, *p* = .006, *η*_p_^2^ = .12]: The PI effect was present in both age groups (*p* < .001), but it was larger in older (mean difference = 112 ms) than in younger (mean difference = 59 ms) adults. Replicating earlier findings (Manard et al., 2014), a higher sensitivity to PI in older adults was found with RTs but not with error rates.

### Diffusion decision model analyses

To test which cognitive factors best explain the behavioral effects, the parameters of six different models were estimated, and a model selection procedure was performed (for more details about the model selection procedure, see Donkin, Brown, & Heathcote, 2011; see also Raftery, 1995; Wasserman, 2000). The parameters of the model that were deemed best by the model selection were interpreted in terms of their cognitive factors.

**Fig. 3:**
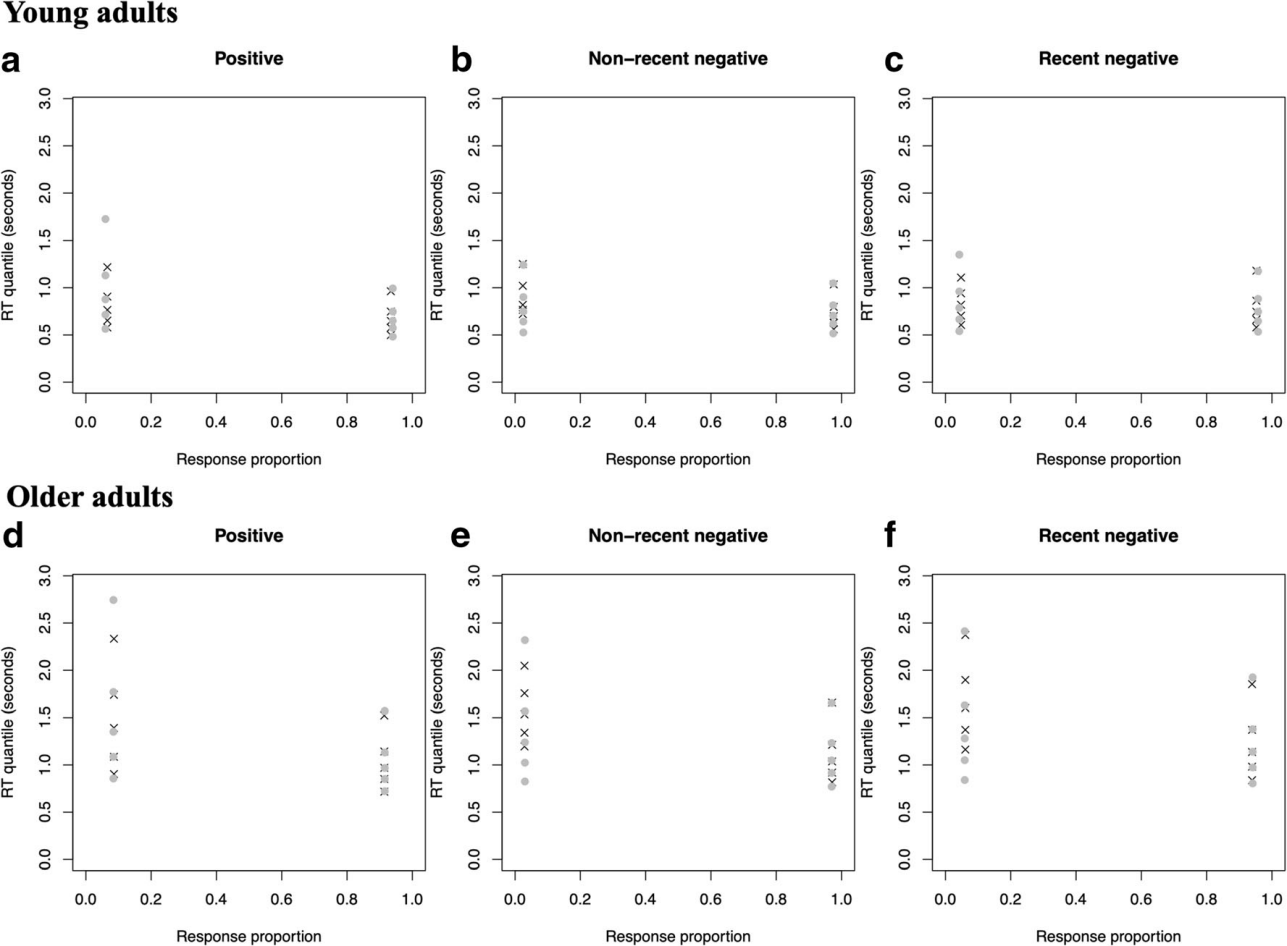
Quantile probability plots showing the best-fitting model, averaged over participants for the positive (A, D), non-recent negative (B, E), and recent negative (C, F) trials, separately for young (top graphs) and older (bottom graphs) adults. Each graph represents the response proportion (*x*-axis) and RTs distributions (represented by five quantiles; *y*-axis). For incorrect (left) and correct (right) responses, the observed and predicted quantiles are represented by crosses and circles, respectively

In Model 1, drift rate and intertrial variability of the drift rate were allowed to vary across the three conditions (positive, non-recent negative, recent negative). All other parameters were held fixed across conditions. In Model 2, drift rate, intertrial variability of the drift rate, boundary separation, and intertrial variability of the starting point were allowed to vary across conditions. All other parameters were held fixed across conditions. In Model 3, drift rate, intertrial variability of the drift rate, boundary separation, intertrial variability of the starting point, nondecision time, and intertrial variability of the nondecision time were allowed to vary across conditions. For these three models, the starting point was fixed at half of the boundary separation. To exclude any potential response bias,^1^ Models 4, 5, and 6 were the same as Models 1, 2, and 3, with the difference that the starting point, fixed across conditions, could differ from half of the boundary separation. We fitted the DDM to the data for each participant separately using maximum likelihood estimation. Likelihood was optimized by using a SIMPLEX search algorithm (Nelder & Mead, 1965) to find the best-fitting parameter values for each model. The diffusion constant was fixed to 0.1, and the measurement scale of RTs was seconds. Because the fit of more complex models (with more free parameters) will be at least as good as the fit of less complex models, the Bayesian information criterion (BIC; Schwarz, 1978) was computed in order to select the model with the best trade-off between model complexity and goodness of fit. For 31 (12 young adults and 19 older adults) of the 63 participants, the best model was Model 1, with drift rate and intertrial variability of the drift rate free to vary across conditions (Table 1). The second best model was the same model with an estimated starting point. This emphasizes that drift rate is really what drives the conditions (for ~85% of the participants). The fit of this model averaged over participants for each condition and age group separately is shown in Fig. 3. Overall, the model fits reasonably well. However, misfits between the data and the model for error responses are observed in each condition and age group, possibly due to the relatively low number of incorrect trials. To conclude, the simplest model provides the better compromise between quality of fit and simplicity. The remaining models also fit the data adequately but introduce unnecessary model complexity. The individual estimated parameter values of the best-fitting model were then used to examine age differences for the main components of the DDM (i.e., drift rate, boundary separation, and nondecision time). The estimated parameter values by age group are provided in Table 2.

**Table 1:** Model selection procedure. Parameters columns depict the different parameter constraints for each model. The *n* Best and Percentage columns, respectively, depict the number of participants and the percentage for that this was the best model, according to Bayesian information criterion values. The Ranking column sorts models (highest ranking = winning model) on the basis of their *n* Best and Percentage values

Parameters						
Model	Free Across Conditions	Fixed Across Conditions	Starting Point	*n* Best	Percentage	Ranking
1	Drift rate Intertrial variability of drift rate	Boundary separation Intertrial variability of starting point Nondecision time Intertrial variability of nondecision time	Unbiased response (half of the boundary separation)	31	49.21%	1
2	Drift rate Intertrial variability of drift rate Boundary separation Intertrial variability of starting point	Nondecision time Intertrial variability of nondecision time	Unbiased response (half of the boundary separation)	4	6.35%	3
3	Drift rate Intertrial variability of drift rate Boundary separation Intertrial variability of starting point Nondecision time Intertrial variability of nondecision time		Unbiased response (half of the boundary separation)	4	6.35%	3
4	Drift rate Intertrial variability of drift rate	Boundary separation Intertrial variability of starting point Nondecision time Intertrial variability of nondecision time	Biased response (fixed across conditions)	23	36.51%	2
5	Drift rate Intertrial variability of drift rate Boundary separation Intertrial variability of starting point	Nondecision time Intertrial variability of nondecision time	Biased response (fixed across conditions)	0	0%	6
6	Drift rate Intertrial variability of drift rate Boundary separation Intertrial variability of starting point Nondecision time Intertrial variability of nondecision time		Biased response (fixed across conditions)	1	1.59%	5

**Table 2:** Estimated parameter values (means and standard errors) for Model 1 in young and older adults for the recent-probes task

Parameters	Young Adults		Older Adults	
	*M*	*SE*	*M*	*SE*
Drift rate				
Positive	0.366	0.025	0.304	0.020
Non-recent negative	0.290	0.017	0.255	0.014
Recent negative	0.230	0.014	0.196	0.011
Boundary separation	0.176	0.010	0.229	0.007
Nondecision time	0.304	0.011	0.455	0.023

**Fig. 4:**
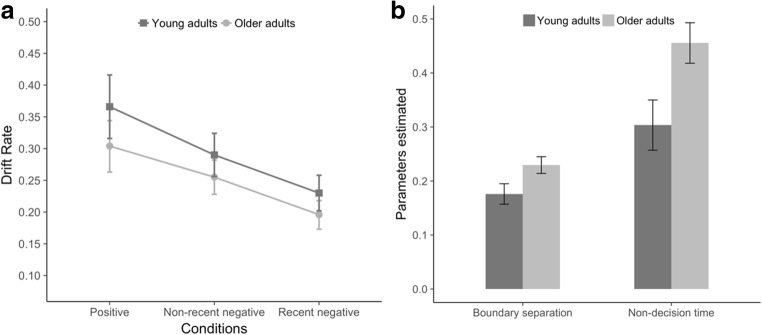
Panel A depicts modeling results of drift rate, averaged over participants and conditions for each age group (error bars represent 95% confidence intervals). Panel B represents modeling results showing age differences for the boundary separation (left) and nondecision time (right) parameters (error bars represent 95% confidence intervals)

The repeated measures ANOVA on drift rate, with condition (three levels: positive, non-recent negative, recent negative) as a within-subjects factor and age (two levels: young adults, older adults) as a between-subjects factor, indicated a significant main effect of condition [*F*(2, 122) = 39.81, *p* < .001, *η*_p_^2^ = .39]. Planned comparisons showed that the drift rate is lower for the recent negative as compared to the non-recent negative condition (*p* < .001), suggesting a PI effect (recent negative condition, mean drift rate = 0.213, *SE* = 0.01; non-recent negative condition, mean drift rate = 0.273, *SE* = 0.01). A significant effect of age [*F*(1, 61) = 5.38, *p* = .024, *η*_p_^2^ = .08] showed a globally lower rate of information processing for older adults (mean drift rate = 0.251, *SE* = 0.01) than for younger adults (mean drift rate = 0.295, *SE* = 0.01); an interaction between condition and age was not observed [*F*(2, 122) = 0.67, *p* = .511, *η*_p_^2^ = .01; Fig. 4A]. Crucially, planned comparisons indicated that the interaction between the PI effect and age for rate of information processing was not significant [*F*(1, 61) = 0.01, *p* = .925, *η*_p_^2^ = .001]. To understand whether the interaction between PI effect and age was not found due to low power or because the null hypothesis was true, we performed a Bayesian ANOVA. This analysis allowed us to quantify the support for the absence of an interaction. The Bayes factor model comparison yielded substantial evidence for the absence of an interaction between PI effect and age. The best model was the additive model (with main effects of both PI and age but with no effect of the interaction PI × Age). This model was preferred by a factor of 5.911 over the full model (with both main effects and an interaction). It is about six times as likely that no interaction was present in the data as that an interaction was present. Regarding the boundary separation parameter, independent-sample *t*-tests revealed a higher boundary separation for older than for younger adults [*t*(61) = 4.39, *p* < .001; Fig. 4B], indicating that older adults needed more information in order to make a decision. Similarly, independent-sample *t*-tests showed a higher nondecision time for older than for younger adults [*t*(61) = 5.09, *p* < .001, Fig. 4B], indicating that older adults took more time for stimulus encoding and response execution.

## Discussion

The aim of the present study was to investigate possible age-related changes in sensitivity to PI in short-term memory. To this end, the performance of young and older adults was compared in a recent-probes task in which probes from a previous trial interfered with the recognition process in the current trial. In addition to traditional analyses, the DDM was applied in order to obtain a more detailed understanding of the latent psychological processes driving age differences in sensitivity to PI.

Inferential analyses on mean performance (RTs and error rates) showed that both young and older adults were sensitive to PI. Non-recent negative trials were performed faster and with fewer errors than recent negative trials. Replicating previous findings (Manard et al., 2014), a higher sensitivity to PI in older adults was observed with RTs, whereas no age effect was found with error rates. Thus, a deficit with age in resistance to PI might be inferred if only RTs were considered as a measure of performance. At the same time, no deficit would be inferred on the basis of accuracy measures. Because the DDM takes into account RTs and response accuracy data simultaneously, it allows for a more comprehensive view concerning the role of age in PI.

First, model selection indicated that the drift rate but not the other main parameters accounted for the behavioral effects observed in the recent-probes task, which can be understood as a difference in processing efficiency. Furthermore, analysis of the main components of the DDM showed a higher boundary separation with age, meaning that older adults adopted a more conservative level of caution than young adults. Finally, longer nondecision times were observed for older than for younger adults, indicating an increase in peripheral processes with age. The results of both boundary separation and nondecision time replicated previous applications of the DDM in the domain of aging (e.g., Ratcliff et al., 2004).

Concerning the drift rate parameter, the results showed a lower processing efficiency for recent negative than for non-recent negative trials. Importantly, the size of this PI effect in short-term memory remained constant with age. Reconciling the findings of both approaches, earlier methodological work had demonstrated that interactions in RTs can be explained by the nonlinear mapping from drift rate to RTs (Wagenmakers, Krypotos, Criss, & Iverson, 2012). Our interpretation of the present results differs from previous studies that had suggested that the inhibitory ability to resist PI declines with age (e.g., Jonides et al., 2000). Our results suggest that the inhibitory functions associated with resistance to PI in a recognition task are not affected by normal aging (for a similar proposal, see Collette et al., 2009). As such, the present results do not match with the inhibitory deficit theory, which argues for a general decline across the different types of inhibitory processes with age (e.g., Lustig et al., 2007). In line with the inhibitory deficit theory, we assume that inhibition is a combination of several functions, rather than a unitary construct (e.g., Friedman & Miyake, 2004; Hasher et al., 1999; Lustig et al., 2007). However, at least the type of inhibition needed to resolve PI in a recognition task seems to be preserved with age. Note that our study focused on PI in a recognition task. Future studies might determine whether our conclusions can be generalized to other types of PI tasks (e.g., retrieval tasks).

Although no increase with age in sensitivity to PI was observed, DDM analyses did reveal overall lower performance (reflected by a smaller overall drift rate) for older than for younger adults. This result suggests an age-related impairment in recognition memory. This is in contrast with a previous study that showed similar drift rates in younger and older adults (Ratcliff et al., 2010). A possible explanation for these inconsistent findings is that our item recognition task and the one implemented by Ratcliff et al. (2010) involved different memory mechanisms. In the item recognition task used by Ratcliff and colleagues, participants had to remember a list of 16 words presented sequentially, which represents a number largely beyond the working memory span. In addition, half of the words were presented twice. After presentation of the list, participants were asked to decide whether or not a test word was part of the study list. Such a task seems to rely more on long-term memory mechanisms, whereas our recent-probes task relies on short-term memory mechanisms.

In summary, through the present study, we aimed to better understand potential age-related changes in sensitivity to PI. Replicating previous studies (e.g., Jonides et al., 2000), inferential analyses indicated an increase in PI with age. This increase is typically explained by a general deficit in inhibitory function with age. The DDM provides a more fine-grained interpretation concerning the latent processing mechanisms underlying performance. First, an analysis of nondecision time and boundary separation confirmed an increase of peripheral processes and level of caution with age. Second, older adults also showed overall lower processing efficiency, indicated by a lower drift rate. This lower drift rate is interpreted as a decline in recognition memory. Crucially, however, the present DDM results did not reveal any evidence for a deficit with age in the general capacity to resist PI. This result seems to indicate that inhibitory abilities to resist PI in short-term memory remain intact in older adults. Further applications of the DDM will help specify exactly which inhibitory processes remain intact with age and which become deficient.

### Author Note

L.V.M. and W.G. contributed equally to this study, and therefore are combined co-senior authors. K.A. was supported by the Belgian Fonds National de la Recherche Scientifique (FNRS) when the study was conducted [grant number FC 4247]. No potential conflict of interest is reported by the authors. The results discussed in this article were presented at the 13th International Conference for Cognitive Neuroscience, 2017, Amsterdam, the Netherlands. No other format was used to share this work.

### Open Practices Statements

None of the data or material for the experiment reported here is available online, but they can be obtained by writing to the first author of this article. This experiment was not preregistered.

## Footnotes

1 We thank an anonymous reviewer for this suggestion.
